# The synaptic action of Degenerin/Epithelial sodium channels

**DOI:** 10.1080/19336950.2018.1495006

**Published:** 2018-08-28

**Authors:** Alexis S. Hill, Yehuda Ben-Shahar

**Affiliations:** Department of Biology, Washington University in St. Louis, St. Louis, USA

**Keywords:** ASIC, DEG/ENaC, neuron, neuronal plasticity, synapse

## Abstract

Degenerin/Epithelial Sodium Channels (DEG/ENaCs) are a large family of animal-specific non-voltage gated ion channels, with enriched expression in neuronal and epithelial tissues. While neuronal DEG/ENaCs were originally characterized as sensory receptor channels, recent studies indicate that several DEG/ENaC family members are also expressed throughout the central nervous system. Human genome-wide association studies have linked DEG/ENaC-coding genes with several neurologic and psychiatric disorders, including epilepsy and panic disorder. In addition, studies in rodent models further indicate that DEG/ENaC activity in the brain contributes to many behaviors, including those related to anxiety and long-term memory. Although the exact neurophysiological functions of DEG/ENaCs remain mostly unknown, several key studies now suggest that multiple family members might exert their neuronal function via the direct modulation of synaptic processes. Here, we review and discuss recent findings on the synaptic functions of DEG/ENaCs in both vertebrate and invertebrate species, and propose models for their possible roles in synaptic physiology.

## Introduction

Members of the DEG/ENaC superfamily of non-voltage-gated, animal-specific cation channels, are expressed in diverse cell types, including neurons [1]. Structure-function studies have revealed that the mature channel is comprised of three independent subunits, which can be either homomeric or heteromeric. Each subunit consists of two transmembrane domains that are connected by a large, extracellular loop that includes several structurally conserved domains [2–5]. Several studies suggest that the extracellular loops play important roles in channel gating by a broad spectrum of extracellular stimuli, including protease activation, mechanical forces, and extracellular chemical ligands [6–8]. The first family members to be identified molecularly were named *Degenerin* (*Deg*) because their mutant alleles were associated with a neuronal degeneration phenotype in the roundworm *Caenorhabditis elegans* [9,10]. Later, mammalian orthologs of DEG proteins were molecularly identified as the elusive amiloride-sensitive epithelial sodium channels (ENaCs), which play an essential role in regulating mammalian blood pressure via salt reabsorption in kidneys [11–13]. Several additional vertebrate family members, namely the Acid Sensing Ion Channels (ASICs; also referred to as Amiloride-sensitive Cation Channels Neuronal; ACCNs), were shown to be activated by low extracellular pH [14–17]. To date, genes that encode putative members of the DEG/ENaC superfamily have been identified in all animal genomes that have been sequenced thus far. Yet, for reasons that are not well understood, the number of independent DEG/ENaC-encoding genes varies dramatically across animal phylogeny, which suggests that the family has expanded and contracted several independent times throughout animal evolution [1,6,18,19].

While the physiological function of DEG/ENaC channels in epithelial tissues is relatively well-understood, the neurophysiological functions of most family members remain unknown. In mammals, neuronally-enriched DEG/ENaC genes primarily belong to the ASIC family branch. To date, five independent ASIC-coding genes have been identified in mammalian genomes (*ASIC1-5*), which are expressed in both central and peripheral neurons, as well as glia [20–25]. In the peripheral nervous system, some ASICs are localized to sensory terminals, where they may play a role in modulating mechanosensation and nociception [26–28]. In the central nervous system, ASICs seem to act as pH sensors, responding to fluctuations in extracellular pH that can be a product of normal neuronal metabolism, acidification from neuronal pathologies, or cell death associated with ischemia and epilepsy [20,29]. In humans, mutations in ASIC genes have been associated with various neurologic and psychiatric disorders [20], including epilepsy [30,31], multiple sclerosis [32,33], and panic disorder [34]. Together, these data suggest that DEG/ENaC channels play an important role in modulating neural functions in humans.

In invertebrate species, DEG/ENaCs have been studied most extensively in the worm *C. elegans* and the fruit fly *Drosophila melanogaster*, which encode 30 and 31 independent DEG/ENaC genes respectively [18,35]. Studies of various family members in these two species indicate that individual DEG/ENaC genes are expressed in diverse cell types, including neurons in both the central and peripheral nervous systems, glia and muscle [36–42]. Some invertebrate DEG/ENaCs that are expressed in the peripheral nervous system have been shown to be gated by mechanosensation or chemical ligands [39,42–45], yet the gating mechanisms and physiological functions for the majority of DEG/ENaCs found in *C. elegans* and *Drosophila* remain mostly unknown.

More recently, several studies suggested that DEG/ENaCs also play an important modulatory role at the synapse in both vertebrate and invertebrate species [41,46–53]. However, the exact cellular and physiological mechanisms that mediate the effects of DEG/ENaCs on synaptic physiology remain poorly understood. Here we review the recent literature on synaptic functions of DEG/ENaC genes, and propose mechanistic models that may explain the observed phenotypes.

## Roles of DEG/ENaCs in behavior

Studies of DEG/ENaC mutant alleles in genetically tractable species such as the mouse, the fly, and the worm revealed the importance of the neuronal functions of DEG/ENaCs to various organismal behavioral phenotypes. For example, studies of *ASIC1a* knockout mice showed that these animals exhibit reduced depression and anxiety related behavior [54], reduced fear behavior [55,56], and increased addiction related behavior [49]. Additionally, *ASIC1a* knockout mice display impaired performance in several learning paradigms, including hippocampal-dependent spatial learning and cerebellar-dependent eyeblink conditioning [57], amygdala-dependent impaired cue and contextual fear conditioning paradigms [51,58–61], and impaired extinction of conditioned taste avoidance [53]. Similarly, mutations in the *C. elegans ASIC-1* gene cause abnormal performance in an associative learning paradigm [62]. Although the precise neurophysiological processes affected by DEG/ENaC signaling remain poorly understood, these data suggest a conserved role for DEG/ENaC signaling in behaviors that are associated with changes in neuronal plasticity, which could be mediated at least in part by DEG/ENaC function at the synapse.

## Roles of DEG/ENaC channels in synaptic physiology and neuronal plasticity

Several studies of the neuronal functions of DEG/ENaCs have suggested that these channels might play a role in mediating synaptic plasticity, which was first highlighted by studies of the function of ASIC1a in the mouse hippocampus. Specifically, electrophysiological recordings in hippocampal slices have shown that the loss of *ASIC1a* has no effect on baseline synaptic transmission, or long-term depression (LTD), but impairs long-term potentiation (LTP) [48,57]. *ASIC1a* knockout mice also display a decreased paired-pulse ratio [63], and impaired EPSP facilitation in response to high frequency stimulation in central synapses [57]. Together, these data suggest that ASIC1a plays a role in mediating hippocampal synaptic plasticity. However, a follow-up study that used a different transgenic strategy to knock out ASIC1a in the nervous system, found no impact of the mutation on hippocampal LTP [64]. Therefore, whether DEG/ENaC-dependent signaling plays a role in long-term neuronal plasticity in the mammalian hippocampus, or under which conditions, remains uncertain. Nevertheless, more recently, several studies have explored the neuronal functions of ASICs in additional brain regions. These studies showed that *ASIC1a* knockout mice also display impaired LTP in the amygdala [50,51] and stronger short-term depression at the calyx of Held synapse [65]. Although knocking out *ASIC* genes in these specific brain regions leads to decreased synaptic activity, studies in other brain regions have found a contrary effect. For example, studies of the mouse insular cortex have found that genetic knockout or pharmacological inhibition of ASIC1a channels decreased the probability of LTD, with no effect on LTP [53], and studies at the rodent neuromuscular junction (NMJ) have found that *ASIC1a* knockout enhances synaptic facilitation [52]. Together, these studies suggest that the effects of DEG/ENaCs on synaptic plasticity vary across neuronal types and brain regions, and can serve to either enhance or dampen synaptic activity.

The effects of mutations in DEG/ENaCs on LTP and LTD suggest that DEG/ENaCs might play a direct role in synaptic physiology. Additional support for a synaptic role for DEG/ENaCs comes from studies demonstrating that some ASIC channels are specifically localized to synaptic sites in mammalian neurons [58], including presynaptic terminals [52] and dendritic spines [57,66,67]. However, it should be noted that other studies have reported a broader subcellular distribution of ASICs in individual central neurons, in contrast to synaptic enrichment [68]. Invertebrate DEG/ENaCs have also been identified at synaptic sites in *C. elegans* and *Drosophila* [47,62], although the majority of localization data in these species has only been assessed by using the overexpression of tagged transgenes, and therefore, the subcellular distribution of native DEG/ENaC channels has yet to be determined in the majority of invertebrate species. In addition to the immunohistochemistry data, immunoprecipitation studies in heterologous expression systems have found that specific ASIC subunits interact with both pre- and postsynaptic proteins, including clathrin and PSD95 [67,69]. Although these molecular interactions are yet to be replicated with natively-expressed proteins *in vivo*, these studies suggest that at least some DEG/ENaCs are localized to either the pre- or the postsynaptic neuronal subcellular compartment.

The precise physiological functions of DEG/ENaC channels at the synapse remain mostly unknown. Electrophysiological studies in cultured neurons have shown that homomeric ASIC1a and heteromeric ASIC1a/ASIC2a channels can mediate an influx of cations, primarily sodium ions, in response to decreased extracellular pH [50,57,58,70]. The sensitivity of these channels to increases in the extracellular concentration of H^+^ suggests the provocative hypothesis that synaptic ASICs might be playing the role of a synaptic pH sensor, which directly responds to synaptic acidification associated with neurotransmitter release. This hypothesis is supported by electrophysiological studies in mouse brain slices, which have shown that synaptic transmission is sufficient to acidify extracellular fluid [71,72], likely due to the highly acidic lumen of synaptic vesicles [73]. However, as we discuss below, several recent studies in vertebrate and invertebrate models suggest that the synaptic roles of DEG/ENaCs might be more complex than as simple synaptic pH sensors.

## DEG/ENaC modulation of presynaptic physiology

In invertebrate and vertebrate model systems, DEG/ENaC channels have been shown to play presynaptic roles, modulating both evoked and spontaneous neurotransmission. Direct evidence for a presynaptic role for DEG/ENaCs was first described in *C. elegans*, where ASIC1 is localized to dopaminergic presynaptic terminals, and *asic-1* mutant worms have been shown to exhibit decreased dopaminergic release [62]. In *Drosophila*, several individual DEG/ENaC subunits have been shown to play presynaptic roles in modulating synaptic plasticity at the larval NMJ, a well-studied excitatory glutamatergic synapse that has many similarities to mammalian central glutamatergic synapses [74,75]. The *Drosophila* NMJ displays robust synaptic homeostatic plasticity, whereby partial blockage of postsynaptic glutamate receptors with either pharmacological or genetic manipulations leads to a homeostatic increase in evoked neurotransmitter release, which subsequently produces “normal” postsynaptic currents in response to stimulation [76]. Recently, a *Drosophila* genetic screen identified three DEG/ENaC-encoding genes, *ppk1, ppk11* and *ppk16*, as presynaptic proteins that are required in motor neurons for this form of homeostatic synaptic plasticity [46,47].

Although currently available genetic tools in vertebrate models do not allow precise anatomical localization of synaptically-enriched DEG/ENaC channels to either pre- or postsynaptic sites, physiological and behavioral data suggest that some synaptic DEG/ENaC channels are likely to exert their function at the presynaptic site. For example, *ASIC1a* knockout in mice has been shown to reduce the paired-pulse ratio [63], a phenotype that typically indicates a presynaptic mechanism [77]. Studies in vertebrates have also revealed that, in addition to their presynaptic impact on evoked release, DEG/ENaCs also play a role in regulating the frequency of spontaneous neurotransmission [52,63,78,79], which further supports a presynaptic role in processes that regulate the rate of synaptic vesicle release [80]. Increased frequency of spontaneous release has been observed in *ASIC1a* knockout mice at peripheral synapses [52] and central synapses in cultured systems [63]. Similarly, pharmacological treatments of hippocampal slices with an ASIC1a antagonist lead to an increase in the frequency of spontaneous neurotransmitter release [79]. Conversely, in brain slice preparations of the amygdala, application of an ASIC1a antagonist decreases the frequency of spontaneous release [78]. How the activity of DEG/ENaC channels might actually regulate spontaneous neurotransmitter release is unclear. Spontaneous neurotransmission has long been appreciated for its role in the development of synaptic connections, yet more recently, has been shown to affect neurotransmission in fully developed animals, including aspects of synaptic plasticity [81]. Therefore, the impact of DEG/ENaCs on spontaneous neurotransmission may be expected to affect both developmental processes as well as synaptic plasticity in adult animals. Together, evidence from invertebrates and vertebrates show that DEG/ENaCs have presynaptic effects on both evoked and spontaneous synaptic transmission.

## DEG/ENaC modulation of postsynaptic physiology

In addition to their function at the presynaptic site, observations that mutations in DEG/ENaC channels also affect processes associated with postsynaptic plasticity, such as LTP and LTD, suggested at least some family members act via postsynaptic processes [48,50,51,53,57]. Particularly, studies have demonstrated that mutations in several ASIC-encoding genes affect the function of postsynaptic glutamate receptors and spine density, further supporting a postsynaptic role for DEG/ENaC channels in modulating synaptic plasticity. Specific examples include the effect of *ASIC1a* mutations on decreased NMDA receptor function in the hippocampus [48], and their association with lower overall dendritic spine density [66]. Conversely, in the nucleus accumbens, *ASIC1A* knockout mice display increased density of dendritic stubby spines, altered glutamate receptor function in the form of increased inward rectification of AMPA currents following evoked release, and an increase in the ratio of AMPA to NMDA receptor currents [49]. Consequently, some ASICs appear to modulate synaptic plasticity via postsynaptic mechanisms, contributing to either facilitation or dampening of synaptic activity.

In addition to data from vertebrate models, physiological and behavioral data from studies with *Drosophila* suggest that DEG/ENaCs contribute to postsynaptic functions in invertebrates as well. Specifically, we recently showed that mutations in the DEG/ENaC-encoding gene *ppk29* lead to decreased amplitude of spontaneous neurotransmission at the larval neuromuscular junction (NMJ), which could be rescued by the expression of *ppk29* specifically in the postsynaptic muscle cells, but not in the presynaptic motor neurons [41]. Conversely, mutations in *ppk1* lead to increased amplitude of spontaneous excitatory neurotransmission at the larval NMJ [47]. Together, these data suggest that in both *Drosophila* and vertebrates, independent postsynaptic DEG/ENaC subunits play opposing roles to either increase or decrease activity at a single synapse.

The exact biophysical and physiological processes associated with the postsynaptic action of *ppk29*, and how it might affect postsynaptic excitatory glutamatergic current influx in response to spontaneous neurotransmission, are still unknown. However, molecular and genetic analyses suggest that the effects of the *ppk29* mutation might be mediated, at least in part, via changes in the relative transcriptional ratio between the major two postsynaptic glutamate receptor subtypes [41]. These data are in agreement with previous findings about the impact of mutations in mammalian ASICs on AMPA and NMDA receptor ratios, as discussed above, and thereby suggests a possible conserved mechanism for the postsynaptic effects of mammalian and insect DEG/ENaCs on synaptic activity through altered glutamate receptor function.

### Models for the synaptic functions of DEG/ENaC channels

Based on our general understanding of the physiological functions of ENaCs in epithelial tissues [1,19,82], it is likely that DEG/ENaCs act as cation channels in the plasma membrane, where they mediate the influx of sodium ions, either as a constitutively open channel, or via extracellular ligand-dependent activation. Indeed, previous reports have shown that ASICs can generate robust currents in response to rapid increases in extracellular acidity [50,57,58,70]. Consistent with this model, DEG/ENaCs appear to have larger effects on neuronal activity following bouts of high firing [48,50,51,57], likely due to the extracellular release of free protons associated with the release of many synaptic vesicles. Thus, although DEG/ENaC channels are not voltage-gated, the activity of some family members is likely to be modulated by neuronal firing rates, and therefore, points to a possible physiological explanation for how they might be modulating neuronal plasticity.

In the mammalian brain, extracellular proton-dependent activation of ASIC channels has been shown to play a role in increasing intracellular calcium levels [66,67], which likely mediates some of the impact of DEG/ENaCs on synaptic plasticity. Although heterologously expressed ASICs can directly mediate calcium influx [14,83], the permeability of ASICs to calcium is low [84,85]. Therefore, the actual mechanism by which DEG/ENaC channel activity modulates calcium currents is not clear. One possible model that may explain the relationship between synaptic DEG/ENaC channels and intracellular changes in neuronal calcium levels might be via the indirect impact of DEG/ENaC-dependent sodium influx, which subsequently leads to increased calcium influx through voltage gated calcium channels [86]. In support of this model, inhibition of voltage gated calcium channels prevents acid induced increases in intracellular calcium [87]. Activation of signaling pathways that trigger calcium release from intracellular stores may also contribute to DEG/ENaC mediated increases in intracellular calcium, although this has not been tested experimentally.

### Model for presynaptic DEG/ENaC functions

**Figure 1.**
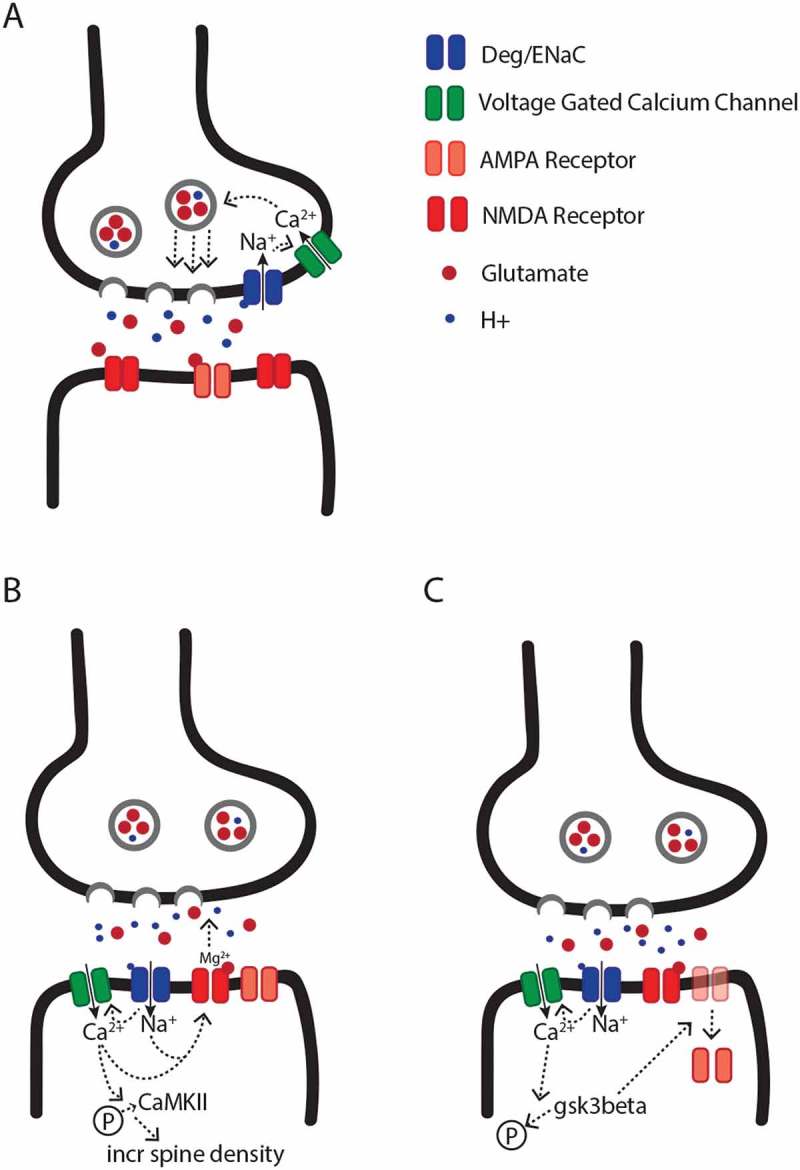
**Models for the putative pre- and postsynaptic functions of DEG/ENaC channels**. (**a**) Model for presynaptic DEG/ENaC function. The lumen of synaptic vesicles is acidic. Therefore, high frequency release of synaptic vesicles leads to an increase in proton concentration in the synaptic cleft. The lower pH leads to opening of presynaptic DEG/ENaCs, followed by a presynaptic sodium influx. Subsequently, local depolarization drives the opening of presynaptic voltage-gated calcium channels, and calcium-dependent synaptic vesicle release. (**b**) Model for postsynaptic DEG/ENaC mediated facilitation of synaptic activity. Upon the presynaptic release of vesicles, the synaptic cleft acidifies, which leads to an influx of cations directly through DEG/ENaC channels or indirectly via voltage-gated calcium channels, and the removal of the extracellular magnesium block from NMDA receptors. Subsequently, the DEG/ENaC-dependent calcium influx also induces the phosphorylation of CaMKII, which increases spine density. (**c**) Model for postsynaptic DEG/ENaC mediated depression of synaptic activity. As in (b), DEG/ENaC-mediated depolarization due to the acidification of the synaptic cleft leads to a calcium influx, which modulates the dephosphorylation and activation of GSK3β, which promotes internalization of postsynaptic AMPA receptors, and subsequently leads to long-term synaptic depression.

The primary model for presynaptic DEG/ENaC function posits that during high frequency synaptic vesicle release, acidification of the extracellular space, and the subsequent induction of DEG/ENaC-dependent influx of sodium ions, modulates the opening of presynaptic voltage-gated calcium channels (Figure 1(a)). Calcium influx through these channels would then be expected to increase calcium dependent synaptic vesicle release [88–90]. This model is consistent with several phenotypes associated with DEG/ENaC knockout animals. For example, studies of dopaminergic neurons in *C. elegans* suggest that mutations in some DEG/ENaC channels lead to decreased calcium influx, which is associated with decreased synaptic vesicle release [62]. Similarly, mutations in some synaptic DEG/ENaC channels have been shown to be associated with decreased glutamate release at the *Drosophila* NMJ during synaptic homeostatic plasticity [46,47], and decreased paired-pulse ratio and frequency of spontaneous release at some mammalian glutamatergic central synapses [63,78].

However, this model does not explain the increased frequency of spontaneous release observed in some brain regions of *ASIC1a* knockout mice [52,63,79]. Although no current physiological and biophysical models may explain this phenotype, it has been suggested that this phenotype may be mediated by non-traditional roles for ASICs, such as direct protein-protein interactions between ASICs and other synaptic components [52,63], which are discussed below.

### Model for postsynaptic DEG/ENaC functions

Empirical studies suggest that the impact of postsynaptic DEG/ENaCs on synaptic physiology can be broadly categorized into two independent pathways, which can lead to either facilitation or depression of synaptic transmission. The primary and most simple model that has been proposed to explain postsynaptic DEG/ENaC-mediated facilitation of synaptic activity suggests that cation influx via DEG/ENaCs leads to either direct or indirect activation of voltage-gated calcium channels, depolarization of the postsynaptic compartment, and the subsequent release of the NMDA receptor magnesium block, which increases NMDA receptor activity [48,57] (Figure 1(b)). In support of this model, ASICs in the rodent hippocampus have been shown to modulate LTP in part by increasing NMDA receptor activity [48,57]. Additionally, ASICs have been shown to modulate intracellular calcium levels, and to increase the levels of phosphorylated calcium/calmodulin-dependent protein kinase II (CaMKII), which has been shown to mediate the effect of ASIC activation on increasing spine density [66]. Whether the impact of ASICs on NMDA receptors and spine density are dependent on each other is unknown.

Postsynaptic DEG/ENaCs have also been shown to play a role in synaptic activity depression. For example, *ASIC1a* knockout mice display a reduced probability of LTD in the insular cortex [53]. Although the exact mechanism for ASIC1a-depedent LTD is not known, it has been hypothesized that direct or indirect DEG/ENaC-dependent modulation of postsynaptic calcium influx leads to the dephosphorylation of glycogen synthase kinase-3β (GSK3β) via phosphatase 1/2A, which drives the internalization of AMPA receptors, and subsequently, depressed synaptic activity (Figure 1(c)) [53]. In support of this model, recent studies suggest that *ASIC1a* is required for the dephosphorylation of GSK3β in the mammalian brain, and the expression of a constitutively active form of GSK3β is sufficient to rescue some of the behavioral and physiological phenotypes associated with *ASIC1a* knockout [53].

DEG/ENaC channels may also directly regulate postsynaptic excitability. For example, a recent intriguing study showed that the postsynaptic activity of ASIC1a is capable of mediating action potentials at the calyx of Held synapse even when postsynaptic glutamate receptors are blocked pharmacologically [65]. However, the authors note that ASIC1a does not appear to have a significant contribution to action potentials under physiological conditions, because neither pharmacological block nor genetic knockout of *ASIC1a* impact action potential generation in the presence of functional glutamate receptors. Nevertheless, these findings suggest that under certain conditions, postsynaptic ASICs may impact synaptic physiology independently of glutamate receptors. Additionally, this study showed that postsynaptic ASIC1a channel activity could modulate short term depression in response to high frequency presynaptic stimulation. Although the mechanism for this effect is unknown, the proposed model suggests that postsynaptic DEG/ENaC channels play a role in a putative retrograde signaling pathway that could modulate presynaptic release probability in response to physiological changes in the postsynaptic cell [65].

The specific mechanistic differences that mediate DEG/ENaC-dependent LTP at some synapses and LTD at others remain mostly unknown. Several non-mutually exclusive explanations include synaptic differences in DEG/ENaC subunit composition, interactions with different classes of voltage gated calcium channels, and differences in intrinsic neuronal activity patterns, which may modulate the time course of acidification of the synaptic cleft. In addition, molecular differences associated with downstream signaling pathways, and the availability of postsynaptic second messengers such as GSK3β, might also contribute to the observed impact of DEG/ENaC channels on glutamatergic neurotransmission.

Here we have presented independent models for synaptic DEG/ENaC function in pre- and postsynaptic compartments, contributing to facilitation or dampening of synaptic activity. However, we suspect these pathways most likely occur in parallel, at least at some synapses. Indeed, at the *Drosophila* NMJ, DEG/ENaCs are present at both pre- and postsynaptic compartments, mediating both increases and decreases in synaptic activity [41,46,47]. Thus, we suspect that in vertebrate synapses as well, DEG/ENaCs may simultaneously modulate several aspects of synaptic physiology.

### Possible contributions of glial DEG/ENaCs to synaptic physiology

Over the past two decades, glial cells have been shown to impact many aspects of neuronal function and synaptic activity [91,92]. Recently, several studies have shown that some DEG/ENaC genes are also expressed in glial cells, including sheath and socket glial cells in *C. elegans* [39,40,93], as well as astrocytes [23,94], microglia [24], NG2 glial cells [95] and oligodendrocytes [22] in rodents. Although the specific cellular and physiological functions of DEG/ENaCs in glia remain mostly unknown, studies suggest that glial ASICs contribute to inward calcium currents in response to decreases in extracellular pH, possibly by acting as receptors for the protons that are co-released with neurotransmitters [22,24,94,95]. In addition, DEG/ENaCs could regulate glial calcium levels, which have been shown to modulate various aspects of neuronal synaptic function, possibly through the release of gliotransmitters, such as glutamate and ATP [96]. Nonetheless, it should be noted that the majority of studies implicating glial DEG/ENaCs in the regulation of synaptic activity in knockout mouse models are confounded by the gene being missing from both neurons and glia. Therefore, the specific division of labor in terms of the specific contributions of neuronal versus glial DEG/ENaCs to synaptic processes is not yet clear.

### Channel activity-independent models for the role of DEG/ENaC proteins in synaptic structure and function

In addition to their possible roles as synaptic ion channels, we would like to consider other possible non-canonical, channel-independent synaptic functions for DEG/ENaC-like proteins. Similar non-canonical neuronal functions have been previously assigned to other classes of ion channels [97–99]. Specifically, as we discuss below, some of the structural features of members of the DEG/ENaC family suggest that these proteins might also contribute to processes associated with synaptic structural integrity and stability.

One of the defining features of all members of the DEG/ENaC protein family is the large extracellular loop [2–5]. Specific structural features of the extracellular loop of several DEG/ENaCs have been hypothesized to resemble peptide neurotoxins that modulate neuronal physiology by directly modifying the functions of voltage-gated sodium and potassium channels [100,101]. In support of this hypothesis, it has been shown that the extracellular loop of ASIC1a can inhibit calcium dependent potassium channels in the BK family, as well as voltage-gated Kv1.3 potassium channels [100,102]. In addition, the inhibitory association between ASIC1a and BK channels has been shown to depend on extracellular pH levels, whereby low pH levels that are sufficient to activate ASICs, are also sufficient to release the inhibition of BK channel activity [100]. In cultured cortical cells, triple knockout of *ASIC1a, ASIC2* and *ASIC3* was shown to increase action potential firing rate and increase the time of the after hyperpolarization in a manner that was blocked by a BK channel inhibitor [102]. Subsequently, it has been hypothesized that some presynaptic effects of ASICs on the frequency of spontaneous neurotransmitter release could be due to interactions between ASICs and other presynaptic ion channels, or direct interactions with the synaptic release machinery [52,63]. However, to date these hypotheses remain theoretical.

In addition to the studies that have implicated protein-protein interactions between DEG/ENaC channels and other neuronal ion channels [100,102], studies in other tissues such as the kidney suggest that ENaC channels physically interact with sodium chloride cotransporters, leading to specific modulations of the activities of both proteins [103]. Together, these studies suggest that physical interactions between DEG/ENaCs and other membrane bound proteins are probably more common than currently appreciated, and likely represent an important facet of DEG/ENaC-dependent physiological processes in general, and modulation of synaptic activity in particular.

Another non-mutually exclusive hypothesis for the possible synaptic functions of DEG/ENaCs relates to the recent discovery that the intracellular domains of DEG/ENaCs may play enzymatic roles to modulate intracellular signaling cascades. Specifically, it was recently shown that independent of its ion channel function, extracellular acidification drives the intracellular C terminus of ASIC1a to bind to and phosphorylate the serine/threonine kinase receptor interaction protein 1 (RIP1), a key mediator of necroptosis [104]. Additionally, bioinformatic analysis of 28 DEG/ENaC protein sequences, including invertebrate and mammalian sequences, identified similarities between the intracellular N terminus and part of a protease domain [105], suggesting a possible enzymatic function for DEG/ENaCs. Studies of other ion channel families have similarly reported interactions with key enzymes in canonical metabotropic signaling pathways. For example, potassium, calcium and transient receptor potential (TRP) channels have been shown to bind to and modulate protein kinases, including CamKII and serine/threonine kinases [97,98]. Although not yet supported by empirical data, these possible interactions between synaptic ion channels, including DEG/ENaCs, and phosphorylation-dependent signaling cascades, could mediate synaptic plasticity via a myriad of downstream pathways, including short term effects on ion channel function and long-term effects on gene expression [106–108].

Another unexplored aspect of DEG/ENaC channels relates to reports that some family members are localized to intracellular membranes, suggesting that instead of responding to extracellular ligands, these channels play a role in intracellular signaling pathways. For example, in addition to its cell membrane localization, ASIC1a has also been observed in mitochondrial membranes of mouse cortical neurons, where it was shown to physically interact with the inner mitochondrial membrane protein adenine nucleotide translocase (ANT) [109]. While ANTs primary function is in ADP/ATP exchange, ANT is also a key mediator of the mitochondria-driven apoptosis pathway in response to oxidative stress [110]. Based on the observed physical interaction between ASIC1a and ANT, ASIC1a is also likely to modulate mitochondrial ADP/ATP exchange in non-oxidative stress conditions. Therefore, because both the pre- and postsynaptic compartments are packed with mitochondria, which are required for proper neuronal development and function via their regulation of ATP supply and calcium homeostasis [111,112], we hypothesize that some DEG/ENaC channels might also affect synaptic physiology via their role in mitochondria.

## Conclusions

In recent years, DEG/ENaCs have emerged as important modulators of neuronal and behavioral plasticity in health and disease. Therefore, understanding their specific action at the synapse is important because they could serve as central targets for drugs that affect neuronal plasticity as a possible solution for diverse cognitive and psychiatric disorders, and for deciphering the basic biological principles that govern synaptic and behavioral plasticity. Although the actual physiological functions of synaptic DEG/ENaCs is still mostly unknown, here we propose several non-mutually exclusive models that might explain such functions, and provide an overview of the empirical data that support at least some of these models. We anticipate that future studies will indicate that individual DEG/ENaC-encoding genes contribute to synaptic physiology and plasticity across neuronal cell types and species via diverse mechanisms and cellular compartments. These studies could be enhanced by a better understanding of the subcellular localization of neuronal DEG/ENaC proteins, especially in the pre- and postsynaptic domains. Because generating antibodies that target membrane bound proteins is notoriously difficult, we expect that the use of CRISPR/Cas9-depedent genome editing approaches in genetically tractable organisms such as the fly, the worm, and the mouse would accelerate this current gap by tagging endogenous DEG/ENaC-encoding genes. Additionally, the use of genome editing for the generation of carefully designed mutant alleles could lead to novel mechanistic insights into the synaptic functions of DEG/ENaCs by directly testing predictions derived from the models we propose here.
